# Supplementary material for: Expanding the CRISPR Toolbox for Engineering Lycopene Biosynthesis in Corynebacterium glutamicum
Source: Microorganisms. 2024 Apr 16;12(4):803. doi: 10.3390/microorganisms12040803 (PMC11052027; doi:10.3390/microorganisms12040803)
Supplement: Supplementary file 1 [file microorganisms-12-00803-s001.zip › microorganisms-2939588-supplementary.pdf]

Supplementary files

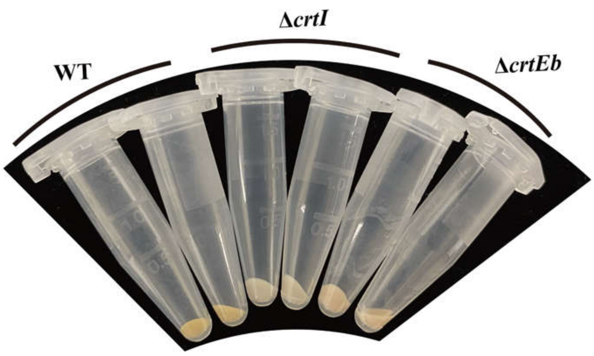

Figure S1 Colour comparison of the WT strain, Δ*crtI* strain, and Δ*crtEb* strain. From left to right strains are WT (*C. glutamicum* ATCC 13032, light yellow), Δ*crtI* (*C. glutamicum* ATCC 13032-Δ*crtI*, white), and Δ*crtEb* (*C. glutamicum* ATCC 13032-Δ*crtEb*, light pink).

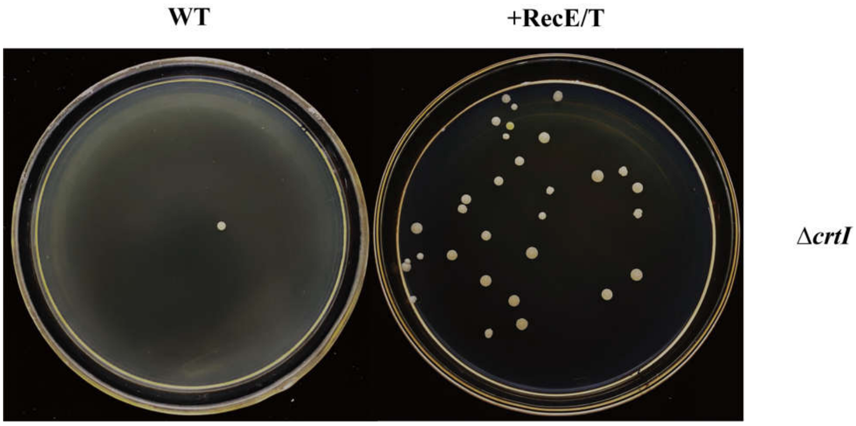

Figure S2 Effect of introduction of recE/T on the number of transformants. Both sides of the plate were introduced CRISPR/MAD7 system for Δ*crtI* in *C. glutamicum* ATCC 13032. The right plate was introduced RecE/T to promote homologous recombination, while the left did not.

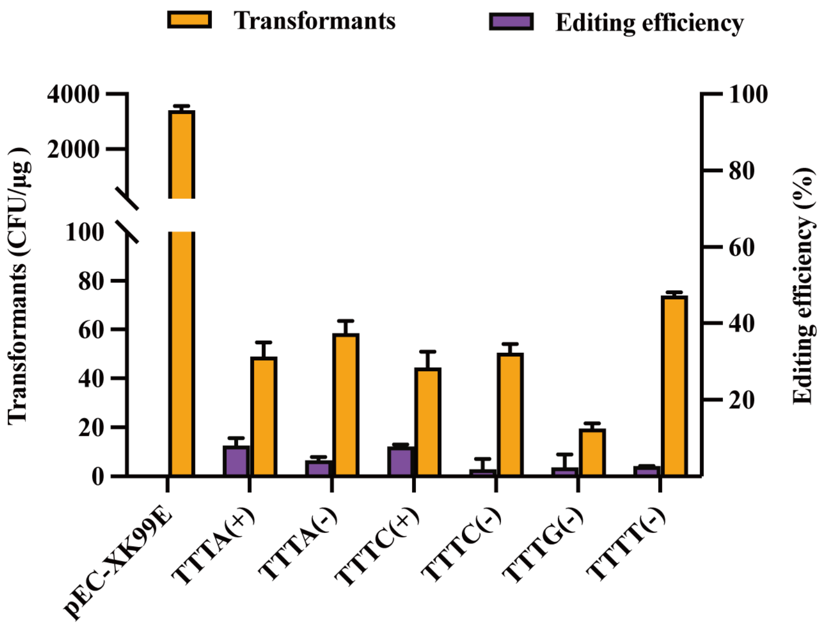

Figure S3 Transformants and editing efficiency of CRISPR/MAD7 using different PAM sequences. The effect of different PAM sequences on the transformants and the editing efficiency of Δ*crtI* by CRISPR/MAD7 system based on pEC-XK99E plasmid and P*trc*-MAD7 in *C. glutamicum* ATCC 13032.

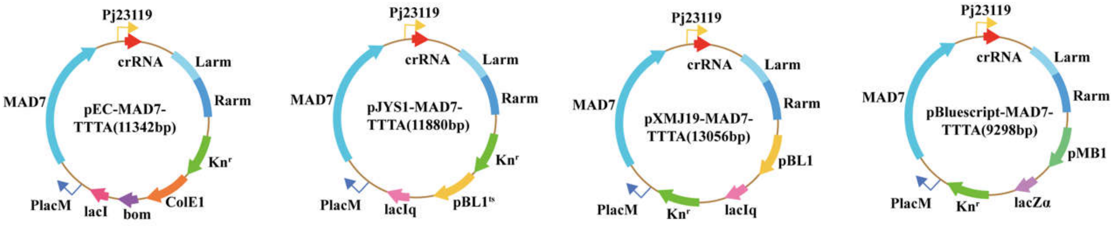

Figure S4 Schematic representation of the different plasmids carrying CRISPR/MAD7 system. All in one CRISPR/MAD7 system constructed using different plasmids, from left to right are based on pEC-XK99E, pJYS1Peftu, pXMJ19 and pBluescript respectively.

WT Δ*upp*

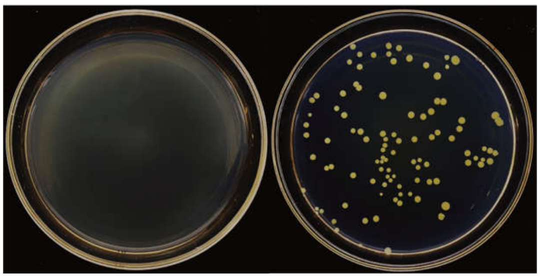

Figure S5 Comparison of the transformants number with knockout of the *upp* gene. The WT (*C. glutamicum* ATCC 13032) and Δupp (*C. glutamicum* ATCC 13032-Δ*upp*) strain on the plates. Both the left and right plate is supplemented with 100 μM 5-fluorouracil. Under normal circumstances, the colonies on the right plate will all be positive.

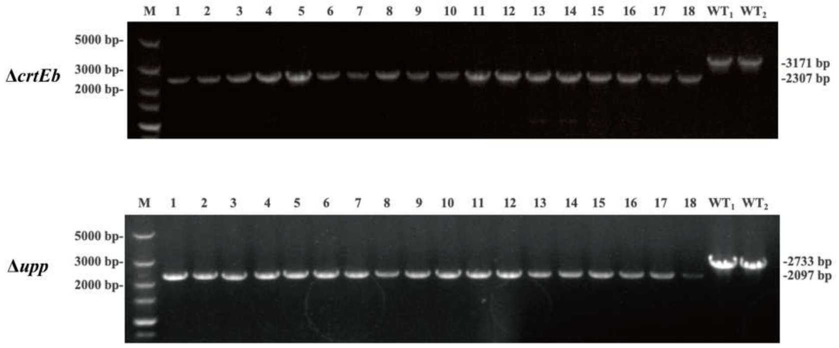

Figure S6 PCR validation of *crtEb* and *upp* gene deletion. From top to bottom, utilizing colony PCR for identification of *crtEb* and *upp* deletion in the genome of *C. glutamicum* ATCC 13032.

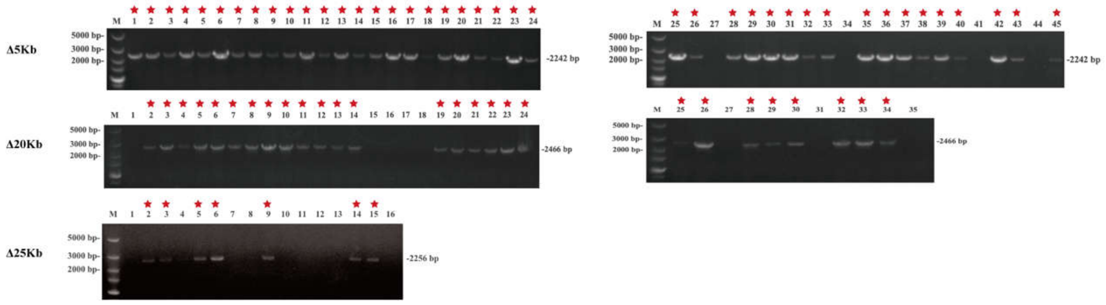

Figure S7 PCR validation of large DNA fragments deletion. From top to bottom, using colony PCR for identification of 5 kb, 20 kb, 25 kb DNA fragments deletion in the genome of *C. glutamicum* ATCC 13032.

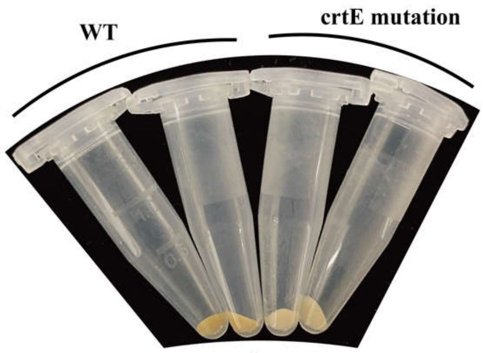

Figure S8 Colour comparison of the WT strain and *crtE* mutation strain. From left to right strains are WT (*C. glutamicum* ATCC 13032, light yellow), *crtE* mutation (*C. glutamicum* ATCC 13032-*crtE* mutation, white).

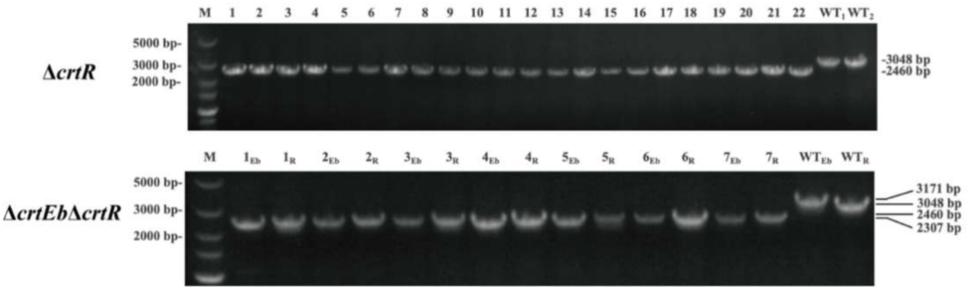

Figure S9 PCR validation of *crtR* gene and double gene deletion. From top to bottom, using colony PCR for identification of Δ*crtR*, Δ*crtEb*Δ*crtR* in *C. glutamicum* ATCC 13032.

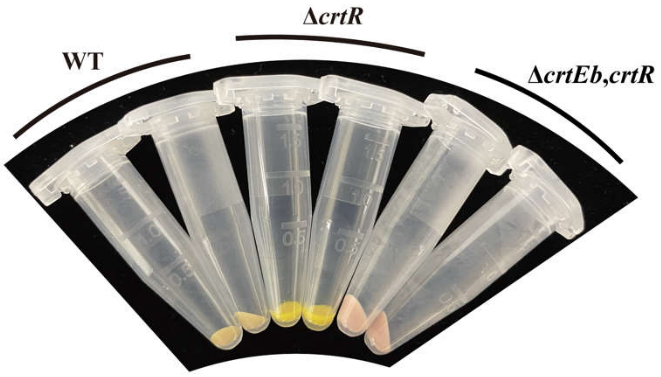

Figure S10 Colour comparison of the WT strain, Δ*crtR* strain, and Δ*crtEb*Δ*crtR* strain. From left to right strains are WT (*C. glutamicum* ATCC 13032, light yellow), Δ*crtR* (*C. glutamicum* ATCC 13032-Δ*crtR*, dark yellow), Δ*crtEb*Δ*crtR* (*C. glutamicum* ATCC 13032-Δ*crtEb*Δ*crtR*, pink).

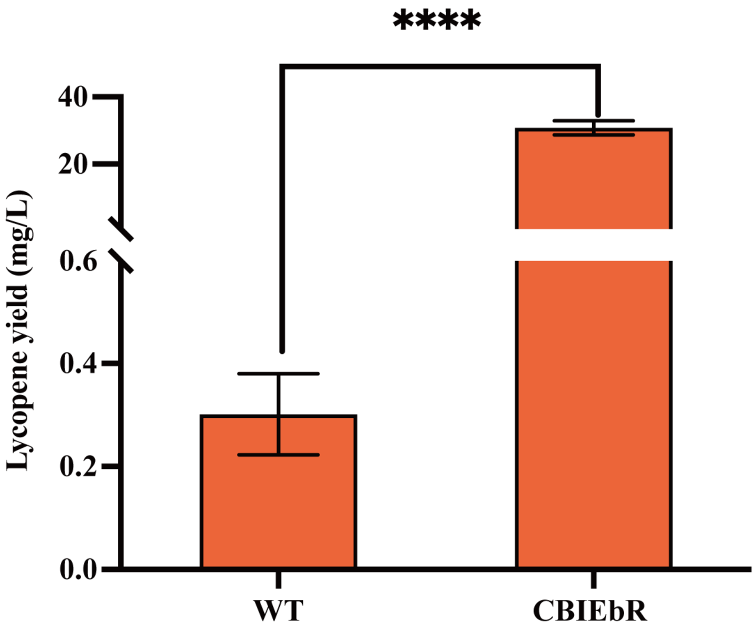

Figure S11 Lycopene production of WT strain and CBIEbR strain. Lycopene yield of WT strain and CBIEbR strain using CGXII medium (40 g/L glucose was added). Data are analyzed using two-tailed t-test, *P < 0.05, **P < 0.01, ***P < 0.001, ****P < 0.0001.

Table S1 Strains and plasmids used in this study.

Strains	properties	Source
C. glutamicum ATCC 13032	wild type	Buy from ATCC
C. glutamicum WT-RecE/T	ATCC 13032 contains pEC-RecE/T plasmid	This study
C. glutamicum WT-RecT	ATCC 13032 contains pEC-recT plasmid	This study
E. coli Top10F′	F′ {laclq Tn10 (Tet^R)} *mcr*A Δ (*mrr-hsd*RMS-*mcr*BC) Φ80 *lac*Z*ΔM15* Δ*lac*X74 *rec*A1 *ara*D139 Δ (*ara-leu*) 7697 *gal*U *gal*K *rpsL* *end*A1 *nup*G	Buy from ATCC
**Plasmids**		
pEC-XK99E	Kn^r, pMB1 *ori*, *bom*, *lacI*, Ptrc, rrnB T1, rrnB T2	Stock in the laboratory
pZ9	Kn^r, p15A *ori*, *rep*, Ptac, rrnB T1, rrnB T2	Stock in the laboratory
pJYS1Peftu	Kn^r, $pBL1^{ts}$ *ori*, pSC101 *ori*, *lacIq*, P*lacM-FnCpf1*, Peftu-*RecT*	Addgene
pXMJ19	Cm^r, P*lacIq*, *lacIq*, pBL1 *ori*, pUC *ori*, rrnB term	Addgene
pBluescript	Amp^r, ColE1 *ori*, f1 *ori* , *lacZα*	Addgene
pEC-RecE/T	Cm^r, pEC-RecE/T derivative for IPTG induced expression of RecE and RecT from Rac prophage from E. coli	Stock in the laboratory
pEC-recT	$Cm^{r,}$ pEC-recT constructed by removing RecE from pEC-RecE/T	This study
pEC-MAD7	Kn^r, pEC-XK99E carrying Ptrc-MAD7	This study
pEC-MAD7-crRNA-TTTA (+)	Kn^r, pEC-XK99E carrying Ptrc-MAD7 and Pj23119-crRNA to targe *crtI* gene, but without carrying homologous arm	This study
pEC-MAD7-TTTA (+)	Kn^r, pEC-XK99E carrying Ptrc-MAD7, Pj23119-crRNA, and homologous arm to targe *crtI* gene	This study
pEC-MAD7-TTTA (-)	Kn^r, pEC-XK99E carrying Ptrc-MAD7, Pj23119-crRNA, and homologous arm to targe *crtI* gene	This study
pEC-MAD7-TTTC (+)	Kn^r, pEC-XK99E carrying Ptrc-MAD7, Pj23119-crRNA, and homologous arm to targe *crtI* gene	This study

pEC-MAD7-TTTC (-)	Kn^r, pEC-XK99E carrying Ptrc-MAD7, Pj23119-crRNA, and homologous arm to targe *crtI* gene	This study
pEC-MAD7-TTTG (-)	Kn^r, pEC-XK99E carrying Ptrc-MAD7, Pj23119-crRNA, and homologous arm to targe *crtI* gene	This study
pEC-MAD7-TTTT (-)	Kn^r, pEC-XK99E carrying Ptrc-MAD7, Pj23119-crRNA, and homologous arm to targe *crtI* gene	This study
pEC-Ptac-MAD7-TTTA (+)	Kn^r, pEC-XK99E carrying Ptac-MAD7, Pj23119-crRNA, and homologous arm to targe *crtI* gene	This study
pEC-Ptuf-MAD7-TTTA (+)	Kn^r, pEC-XK99E carrying Ptuf-MAD7, Pj23119-crRNA, and homologous arm to targe *crtI* gene	This study
pEC-PlacM-MAD7-TTTA (+)	Kn^r, pEC-XK99E carrying PlacM-MAD7, Pj23119-crRNA, and homologous arm to targe *crtI* gene	This study
pXMJ19-MAD7-TTTA (+)	Kn^r, pXMJ19 carrying PlacM-MAD7, Pj23119-crRNA, and homologous arm to targe *crtI* gene	This study
pBluescript-MAD7-TTTA (+)	Kn^r, pBluescript carrying PlacM-MAD7, Pj23119-crRNA, and homologous arm to targe *crtI* gene	This study
pJYS1-MAD7-TTTA (+)	Kn^r, pJYS1Peftu carrying PlacM-MAD7, Pj23119-crRNA, and homologous arm to targe *crtI* gene	This study
pJYS1-MAD7-TTTA (-)	Kn^r, pJYS1Peftu carrying PlacM-MAD7, Pj23119-crRNA, and homologous arm to targe *crtI* gene	This study
pJYS1-MAD7-TTTC (+)	Kn^r, pJYS1Peftu carrying PlacM-MAD7, Pj23119-crRNA, and homologous arm to targe *crtI* gene	This study
pJYS1-MAD7-TTTC (-)	Kn^r, pJYS1Peftu carrying PlacM-MAD7, Pj23119-crRNA, and homologous arm to targe *crtI* gene	This study
pJYS1-MAD7-TTTG (-)	Kn^r, pJYS1Peftu carrying PlacM-MAD7, Pj23119-crRNA, and homologous arm to targe *crtI* gene	This study
pJYS1-MAD7-TTTT (-)	Kn^r, pJYS1Peftu carrying PlacM-MAD7, Pj23119-crRNA, and homologous arm to targe *crtI* gene	This study

pJYS1-MAD7-CTTT	Kn^r, pJYS1Peftu carrying PlacM-MAD7, Pj23119-crRNA, and homologous arm to targe *crtI* gene	This study
pJYS1-MAD7-CTTG	Kn^r, pJYS1Peftu carrying PlacM-MAD7, Pj23119-crRNA, and homologous arm to targe *crtI* gene	This study
pJYS1-MAD7-CTTA	Kn^r, pJYS1Peftu carrying PlacM-MAD7, Pj23119-crRNA, and homologous arm to targe *crtI* gene	This study
pJYS1-MAD7-CTTC	Kn^r, pJYS1Peftu carrying PlacM-MAD7, Pj23119-crRNA, and homologous arm to targe *crtI* gene	This study
pJYS1-MAD7-TTTC-Δ*crtEb*	Kn^r, pJYS1Peftu carrying PlacM-MAD7, Pj23119-crRNA, and homologous arm to targe *crtEb* gene	This study
pJYS1-MAD7-TTTG-Δ*crtEb*	Kn^r, pJYS1Peftu carrying PlacM-MAD7, Pj23119-crRNA, and homologous arm to targe *crtEb* gene	This study
pJYS1-MAD7-CTTT-Δ*crtEb*	Kn^r, pJYS1Peftu carrying PlacM-MAD7, Pj23119-crRNA, and homologous arm to targe *crtEb* gene	This study
pJYS1-MAD7-CTTG-Δ*crtEb*	Kn^r, pJYS1Peftu carrying PlacM-MAD7, Pj23119-crRNA, and homologous arm to targe *crtEb* gene	This study
pJYS1-MAD7-CTTA-Δ*crtEb*	Kn^r, pJYS1Peftu carrying PlacM-MAD7, Pj23119-crRNA, and homologous arm to targe *crtEb* gene	This study
pJYS1-MAD7-CTTC-Δ*crtEb*	Kn^r, pJYS1Peftu carrying PlacM-MAD7, Pj23119-crRNA, and homologous arm to targe *crtEb* gene	This study
pJYS1-MAD7-TTTC-Δ*upp*	Kn^r, pJYS1Peftu carrying PlacM-MAD7, Pj23119-crRNA, and homologous arm to targe *upp* gene	This study
pJYS1-MAD7-TTTG-Δ*upp*	Kn^r, pJYS1Peftu carrying PlacM-MAD7, Pj23119-crRNA, and homologous arm to targe *upp* gene	This study
pJYS1-MAD7-CTTT-Δ*upp*	Kn^r, pJYS1Peftu carrying PlacM-MAD7, Pj23119-crRNA, and homologous arm to targe *upp* gene	This study
pJYS1-MAD7-CTTG-Δ*upp*	Kn^r, pJYS1Peftu carrying PlacM-MAD7, Pj23119-crRNA, and homologous arm to targe *upp* gene	This study

pJYS1-MAD7-CTTA-Δ*upp*	Kn^r, pJYS1Peftu carrying PlacM-MAD7, Pj23119-crRNA, and homologous arm to targe *upp* gene	This study
pJYS1-MAD7-CTTC-Δ*upp*	Kn^r, pJYS1Peftu carrying PlacM-MAD7, Pj23119-crRNA, and homologous arm to targe *upp* gene	This study
pJYS1-MAD7-TTTC-Δ5 kb	Kn^r, pJYS1Peftu carrying PlacM-MAD7, Pj23119-crRNA, and homologous arm to targe 5 kb gene	This study
pJYS1-MAD7-TTTC-Δ20 kb	Kn^r, pJYS1Peftu carrying PlacM-MAD7, Pj23119-crRNA, and homologous arm to targe 20 kb gene	This study
pJYS1-Cpf1-TTTC-Δ20 kb	Kn^r, pJYS1Peftu carrying PlacM-Cpf1, Pj23119-crRNA, and homologous arm to targe 20 kb gene	This study
pJYS1-MAD7-TTTC-Δ25 kb	Kn^r, pJYS1Peftu carrying PlacM-MAD7, Pj23119-crRNA, and homologous arm to targe 25 kb gene	This study
pJYS1-MAD7-TTTC-*crtE*	Kn^r, pJYS1Peftu carrying PlacM-MAD7, Pj23119-crRNA, and homologous arm to targe *crtE* gene	This study
pJYS1-MAD7-TTTC-Δ*crtR*	Kn^r, pJYS1Peftu carrying PlacM-MAD7, Pj23119-crRNA, and homologous arm to targe *crtR* gene	This study
pJYS1-MAD7-TTTC-Δ*crtEb*Δ*crtR*	Kn^r, pJYS1Peftu carrying PlacM-MAD7, Pj23119-crRNA array, and homologous arm to targe *crtEb* and *crtR* gene	This study
pZ9-*crtE-crtB-crtI*	Kn^r, pZ9 carrying one copy of the *crtE-crtB-crtI* gene	This study
pZ9-*cg0722-crtB-crtI*	Kn^r, pZ9 carrying one copy of the *cg0722-crtB-crtI*	This study
pZ9-*crtE- cg0722-crtB-crtI*	Kn^r, pZ9 carrying one copy of the *crtE-cg0722-crtB-crtI*	This study
pZ9-*idsA-idi-crtB-crtI*	Kn^r, pZ9 carrying one copy of the *idsA-idi-crtB-crtI*	This study

Table S2 Primers used in this study.

Primers	DNA Sequence (5'to3')	Reference
P1	GAGAGAAGATTTTCAGCCTGATACAGAT	To amplify pEC-XK99E backbone fragment
P2	TCTGTTTCCTGTGTGAAATTGTTATCCGCTCACAATTC	

P3	TTTCACACAGGAAACAGAATATGAACAACGGC	To clone pEC-MAD7
P4	CAGGCTGAAAATCTTCTCTCT	
P5	ATTAATGCAGCTGGCACGACA	To identify pEC-MAD7
P6	TAGGTGGAGGTATCGGAGAAA	
P7	TCCGATACCTCCACCTACGAA	
P8	CCGCTCGAGGGATGAATGTCAGCTACTGGG	To amplify crRNA-TTTA (+) fragment
P9	TCCCCGCGGCGATCCTCATCCTGTCTCTTG	
P10	CGAAGCGGCACTTTAGTGCAT	To identify pEC-MAD7-crRNA-TTTA (+)
P11	AAGGCTCAGTCGAAAGACTGGGCCTTTCGTTTTATGATGTTATACGGGATAAGAAA	To amplify Δ*crtI* homology arm
P12	AGCGCTCAAATAAAACGAAAGGCTCAGTCG	
P13	ATCGATGATAGGGATCAAAATTCACGAATTCTTTCTGTAGATGCT	
P14	ATTTTGATCCCTATCATCGAT	
P15	GGTCGCGATGCCTGCTTTGTTCTACGACATC	
P16	GTTTTTAAATCGACGGCGGCATCGAGTGCGTC	To clone pEC-MAD7-TTTA (+)
P17	CGTTTTATTTGAGCGCTAGTGCTAAAAGTGCAGAAGTG	
P18	CCCAGGAGGATTAATACCGCTAGCGCTCAAATAAAACGAAAGGCTCAGTCG	To clone pEC-MAD7-TTTA (-)
P19	GGTATTAATCCTCCTGGGCGATCTACAAGAGTAGAAATTAAAAAGGTCTTTTGACACT	
P20	TTTAATTTCTACTCTTGTAGATGATGGGATACCGGACCTTCTTCTAGCGCTCAAATAA	To clone pEC-MAD7-TTTC (+)
P21	AGCGCTAGAAGAAGGTCCGGTATCCCATCATCTACAAGAGTAGAAATTAAAAAGGTCTT	
P22	AGCGCTAGGTATCCAGCAGGTGCAACGCCATCTACAAGAGTAGAAATTAAAAAGGTCTT	To clone pEC-MAD7-TTTC (-)
P23	TTTAATTTCTACTCTTGTAGATGGCGTTGCACCTGCTGGATACCTAGCGCTCAAATAA	

P24	TTTAATTTCTACTCTTGTAGATCACTTTTCTGTTGTGAAGCAACTAGCGCTCAAATAA	To clone pEC-MAD7-TTTG (-)
P25	AGCGCTAGTTGCTTCACAACAGAAAAGTGATCTACAAGAGTAGAAATTAAAAAGGTCTT	
P26	TTTAATTTCTACTCTTGTAGATAGGCGGCAATGGTTCGGGCAGCTAGCGCTCAAATAA	To clone pEC-MAD7-TTTT (-)
P27	AGCGCTAGCTGCCCGAACCATTGCCGCCTATCTACAAGAGTAGAAATTAAAAAGGTCTT	
P28	GCTAGCTCAGTCCTAGGTATAAT	To identify pEC-MAD7-TTTN (+)
P29	CATCGGCTCGTATAATGATGAACAACGGCACCAACAAC	To clone pEC-Ptac-MAD7-TTTA (+)
P30	CATTATACGAGCCGATGATTAATTGTCAACAGCTCATTTCAGAATATTTGCCAG	
P31	GATATCCTTCAGGATCTGGCG	To identify pEC-Ptac-MAD7-TTTA (+)
P32	GTCCAGGAGGACATACAATGAACAACGGCACCAACAAC	To clone pEC-Ptuf-MAD7-TTTA (+)
P33	TGTATGTCCTCCTGGACTTCGCAGCTCATTTCAGAATATTTGCCAG	
P34	TCTTTGATGATGCCGTTCTTCACGATG	To identify pEC-Ptuf-MAD7-TTTA (+)
P35	GTGTGGTACCATGTGTGGAATTGGAAAGGACTTGAACGATGAACAACGGCACCAACAAC	To clone pEC-PlacM-MAD7-TTTA (+)
P36	ACACATGGTACCACACGATGATTAATTGTAAACAGCCAGCTCATTTCAGAATATTTGCC	
P37	TCCAATCGATATCATCGATGGAG	To identify pEC-PlacM-MAD7-TTTA (+)
P38	GGTGCCGTTGTTCATCGTTCAAGTCCTTTCCAATT	To clone pJYS1-MAD7-TTTA (+)
P39	CTAGGACTGAGCTAGCTGTCAACAGCCTGGCGGTGTAATGCA	
P40	GCTAGCTCAGTCCTAGGTATAAT	
P41	ATGAACAACGGCACCAACAA	
P42	GCAAAAACAACGAACCACACT	To identify pJYS1-MAD7-TTTA (+)
P43	GACTGAGCTAGCTGTCAACGAAATGACCGACCAAGCGAC	To clone pXMJ19-MAD7-TTTA (+)

P44	TTTCTCTTTGCGCTTGCGGCCAGGGTGGTT TTTCTTTTCACCAGT	
P45	CGCAAGCGCAAAGAGAAAGCAG	
P46	TTGACAGCTAGCTCAGTCCTAGG	
P47	AACCCCGTTCGATATTTTGTG	To identify pXMJ19-MAD7-TTTA (+)
P48	TTTCTCTTTGCGCTTGCGCACATTTCCCCG AAAAGTGC	To clone pBluescript-MAD7-TTTA (+)
P49	GACTGAGCTAGCTGTCAACTGTCAGACCA AGTTTACTCATATATAC	
P50	GTAACAGGATTAGCAGAGCGA	To identify pBluescript-MAD7-TTTA (+)
P51	GTAGAACAAAGCAGGCAAGAATTTAAAT AAAACGAAAGGCTCAGTCG	To clone pJYS1-MAD7-TTTA (-) / TTTC (+) / TTTC (-) / TTTT (-) / TTTG (-)
P52	CTAGGACTGAGCTAGCTGTCAACAGCCTG GCGGTGTAATGCACC	
P53	ATGGGATACCGGACCTTCTCTAGCGCTCA AATAAAACGAAAGGC	To clone pJYS1-MAD7-CTTT
P54	AGGTCCGGTATCCCATCGATCTACAAGAG TAGAAATTAAAAAGGTCTTTTGACACTAG	
P55	AGCCATCACGTGCTAAAAGCTAGCGCTCA AATAAAACGAAAGGC	To clone pJYS1-MAD7-CTTG
P56	TTAGCACGTGATGGCTGGATCTACAAGAG TAGAAATTAAAAAGGTCTTTTGACACTAG	
P57	GATGCATTGTGGGGGCGGGATCTACAAGA GTAGAAATTAAAAAGGTCTTTTGACACTA G	To clone pJYS1-MAD7-CTTA
P58	CCCCCACAATGCATCAACTAGCGCTCAAA TAAAACGAAAGGC	
P59	CTGTCTTCCCGACCCACTACTCTAGCGCTC AAATAAAACGAAAGGC	To clone pJYS1-MAD7-CTTC
P60	TGGGTCGGGAAGACAGATCTACAAGAGT AGAAATTAAAAAGGTCTTTTGACACTAG	
P61	TACGGAAGGATCTGAGGTTCT	To identify pJYS1-MAD7-YTTN
P62	AAAGGCTCAGTCGAAAGACTGGGCCTTTC GTTTTATGGGTGTAGGAATACCCATGTGTT	To amplify homology arm and crRNA for Δ*crtEb*
P63	GAGGACTACGGCTTTTCTGGCTCA	
P64	CCAGAAAAGCCGTAGTCCTCTGCTTTTAT CCGATCAGTTCTG	

P65	TAAGCAAGACGCCCAATTTCG	
P66	TGCTGAATAAGCAATCACTCTAGCGCTCAAATAAAACGAAAGGCTCAGTCGAAAGACTG	
P67	TGGGCGTCTTGCTTAAGAATTTAAATAAAACGAAAGGCTCAGTCG	To clone pJYS1-MAD7-TTTC-Δ*crtEb*
P68	GAGTGATTGCTTATTCAGCACCATCTACAAGAGTAGAAATTAAAAAGGTCTTTTGACAC	
P69	CATTACAGATGCCAGTTGCTAGCGCTCAAATAAAACGAAAGGCTCAGTCGAAAGACTG	To clone pJYS1-MAD7-TTTG-Δ*crtEb*
P70	CAACTGGCATCTGTAATGTTCATCTACAAGAGTAGAAATTAAAAAGGTCTTTTGACAC	
P71	TGACACTCTCAGTGCTCTAGCGCTCAAATAAAACGAAAG	To clone pJYS1-MAD7-CTTT-Δ*crtEb*
P72	AGCACTGAGAGTGTCAGCCATATCTACAAGAGTAGAAATTAAAAAGGTCTTTTGACAC	
P73	GCAGTACAGGATGTTAATGCTAGCGCTCAAATAAAACGAAAGGCTCAGTCGAAAGACTG	To clone pJYS1-MAD7-CTTG-Δ*crtEb*
P74	CATTAACATCCTGTACTGCTCATCTACAAGAGTAGAAATTAAAAAGGTCTTTTGACAC	
P75	CGTATAACATCGCCATGTCTAGCGCTCAAATAAAACGAAAGGCTCAGTCGAAAGACTG	To clone pJYS1-MAD7-CTTA-Δ*crtEb*
P76	ACATGGCGATGTTATACGGGAATCTACAAGAGTAGAAATTAAAAAGGTCTTTTGACAC	
P77	GTCTGCATTAACATCCTGCTAGCGCTCAAATAAAACGAAAGGCTCAGTCGAAAGACTG	To clone pJYS1-MAD7-CTTC-Δ*crtEb*
P78	CAGGATGTTAATGCAGACCGGATCTACAAGAGTAGAAATTAAAAAGGTCTTTTGACAC	
P79	AAGTCATGGTTCAACCTCGG	To identify pJYS1-MAD7-YTTN-Δ*crtEb*
P80	TGCAGCAGCCAACGACCTCCTAGCGCTCAAATAAAACGAAAGGCTCAGTCGAAAGACTG	To amplify homology arm and crRNA for Δ*upp*
P81	CGAAAGGCTCAGTCGAAAGACTGGGCCTTTCGTTTTATAAGCCCACTGTGTTAAACCGC	
P82	TGCCCTTAGAAACTTAGCTTCACATGTTAAATCATTGCCG	

P83	GAAGCTAAGTTTCTAAGGCATTACGGAAAATTCTTGCCC	
P84	CCGAGACACTGACGGGATTCA	
P85	CCGTCAGTGTCTCGGAGAATTTAAATAAAACGAAAGGCTCAGTCGAAAGAC	To clone pJYS1-MAD7-TTTC-Δ*upp*
P86	TCGTTGGCTGCTGCACGATCTACAAGAGTAGAAATTAAAAAGGTCTTTTGACAC	To clone pJYS1-MAD7-TTTC-Δ*upp*
P87	TCGATGGTGGCGGTAACATCTACAAGAGTAGAAATTAAAAAGGTCTTTTGACAC	To clone pJYS1-MAD7-TTTG-Δ*upp*
P88	TACCGCCACCATCGACCCACTAGCGCTCAAATAAAACGAAAGGCTCAGTCGAAAGACTG	To clone pJYS1-MAD7-TTTG-Δ*upp*
P89	TCGTTGGCTGCTGCACGGATCTACAAGAGTAGAAATTAAAAAGGTCTTTTGACAC	To clone pJYS1-MAD7-CTTT-Δ*upp*
P90	TGCAGCAGCCAACGACCTCTAGCGCTCAAATAAAACGAAAGGCTCAGTCGAAAGACTG	To clone pJYS1-MAD7-CTTT-Δ*upp*
P91	CCGGTGATCGTCTCTCTAGCGCTCAAATAAAACGAAAGGCTCAGTCGAAAGACTG	To clone pJYS1-MAD7-CTTG-Δ*upp*
P92	AGAGACGATCACCGGCATCTCATCTACAAGAGTAGAAATTAAAAAGGTCTTTTGACAC	To clone pJYS1-MAD7-CTTG-Δ*upp*
P93	TGGCAGCGCCTCAACTAGCGCTCAAATAAAACGAAAGGCTCAGTCGAAAGACTG	To clone pJYS1-MAD7-CTTA-Δ*upp*
P94	TTGAGGCGCTGCCACAGGATCATCTACAAGAGTAGAAATTAAAAAGGTCTTTTGACAC	To clone pJYS1-MAD7-CTTA-Δ*upp*
P95	AGTACCTTCAGCCATGCTAGCGCTCAAATAAAACGAAAGGCTCAGTCGAAAGACTG	To clone pJYS1-MAD7-CTTC-Δ*upp*
P96	CATGGCTGAAGGTACTCGCCTATCTACAAGAGTAGAAATTAAAAAGGTCTTTTGACAC	To clone pJYS1-MAD7-CTTC-Δ*upp*
P97	CAACAACACCACCTCCTTGT	To identify pJYS1-MAD7-YTTN-Δ*upp*
P98	AGGCTCAGTCGAAAGACTGGGCCTTTCGTTTTATGTCTCAATTTCTGGATTGAGCCTG	To amplify Δ5 kb homology arm and crRNA
P99	ACTCGCGGGCAATAACGTAGAGCGATAGTCTGCGGG	To amplify Δ5 kb homology arm and crRNA
P100	TTATTGCCCGCGAGTCTGCTG	To amplify Δ5 kb homology arm and crRNA
P101	GATTTGCTTTGCGGTATCCCAAC	To amplify Δ5 kb homology arm and crRNA
P102	CACCCATGACCTCTCTAGCGCTCAAATAAAACGAAAGGCTCAGTCGAAAGACTGGG	To amplify Δ5 kb homology arm and crRNA
P103	TACCGCAAAGCAAATCAGAATTTAAATAAAACGAAAGGCTCAGTCG	To clone pJYS1-MAD7-TTTC-Δ5 kb

P104	AGAGGTCATGGGTGTGTAGTAATCTACAAGAGTAGAAATTAAAAAGGTCTTTTGACAC	
P105	CAACTCACCGACAATGCCGAAA	To identify pJYS1-MAD7-TTTC-Δ5 kb
P106	CGGTCTGAATGGTGTTCATCGG	
P107	TGGTCATGCACCACATTGATGAGCAC	To amplify Δ 20kb homology arm
P108	TTCGCCGATGCCGTAGATGAATGAGGTTTTACCG	
P109	ATCTACGGCATCGGCGAAGGTCAAGATCAGGGTTATTAACCATTTTCTGCGC	
P110	ATTCGTCTCACTGATACTCGCGCCAA	
P111	GGCTCAGTCGAAAGACTGGGCCTTTCGTTTTATTGGTCATGCACCACATTGATGAGCAC	To clone pJYS1-MAD7-TTTC-Δ20 kb
P112	CGCGAGTATCAGTGAGACGAATAGAATTTAAATAAAACGAAAGGCTCAGTCG	
P113	GAAGAAGTACACGTTCTTGGTGAGTGC	To identify pJYS1-MAD7-TTTC-Δ20 kb
P114	AATGACCAGATGCGACTGCCCAATA	
P115	AGAGGTCATGGGTGTGTAGTAATCTACAAGAGTAGAAATTAAAAAGGTCTTTTGACAC	To clone pJYS1-Cpf1-TTTC-Δ20 kb
P116	CGCGAGTATCAGTGAGACGAATAGAATTTAAATAAAACGAAAGGCTCAGTCG	
P117	TTAGCGGACTTGAAGTCCTTCTGCA	To identify pJYS1-Cpf1-TTTC-Δ20 kb
P118	CCGACAAGAAGTTCTTCGCAAAGCT	
P119	TCTCAGACATCAACAAACGCCCCA	To amplify Δ25 kb homology arm
P120	GCGGAATTGTGGGACACCGTATCTGTGTTGGTCTCTATTATGCCGGGA	
P121	ATACGGTGTCCCACAATTCCGC	
P122	AAGCAATAGGATCTGTGTGCTGGT	
P123	CACACAGATCCTATTGCTTAGAATTTAAATAAAACGAAAGGCTCAGTCG	To clone pJYS1-MAD7-TTTC-Δ25 kb
P124	TTACTTCTTCAGCGATCTAGCGCTCAAATAAAACGAAAGGCTCAGTCGAAAGACTGGGC	
P125	GATAACTCACCGTTTGATCCGG	

P126	CTTTAATGATGGGGACATGCGC	To identify pJYS1-MAD7-TTTC-Δ25 kb
P127	TAAGCAGTCCATTAGATTCTAGCGCTCAAATAAAACGAAAGGCTCAGTCGAAAGACTGG	To clone pJYS1-MAD7-TTTC-*crtE*
P128	TCTAATGGACTGCTTATGGATCTACAAGAGTAGAAATTAAAAAGGTCTTTTGACACTAG	
P129	CTGAAGGGCCTCCTTTTTGTTATCCG	To clone pEC-recT
P130	AAGGAGGCCCTTCAGATGACTAAGCAACCACCAATCGCAAA	
P131	CTGTTTGCGCGTCATAACTTC	To identify pEC-recT
P132	ATGGACAATGGCATGACAATCAC	To amplify *crtE* sequence
P133	CTAAGATTTGCGGCTGGCTAGT	
P134	TGGGTGGCAGCAAGAAAAAGATTAACCATAAGCAGTCCATTAGATTGTGCCCAATCTAG	59 bp, corresponding to the mutation of PAM (TTTC) on the *crtE* gene
P135	AAGGTCAAGGCGCGCGAAGATTTGATGGGTGGCAGCAAGAAAAAGATTAACCATAAGCAGTCCATTAGATTGTGCCCAATCTAGATGCTCTGGATCGCG	100 bp, corresponding to the mutation of PAM (TTTC) on the *crtE* gene
P136	TCGGCTGCGTCACCAAAAGTA	To amplify Δ*crtR* homology arm
P137	CACTTTAATTAACGTAGATGGTCATAGTAATCACCTTTTTCTACAAAGTCAGATAAAG	
P138	ATGACCATCTACGTTAATTAAAGTGTGCAGAG	
P139	AGTCAAAGCGCTGAAGACTTGT	
P140	TACTTTTGGTGACGCAGCCGAATAAAACGAAAGGCCCAGTCT	To clone pJYS1-MAD7-TTTC-Δ*crtR*
P141	ACAAGTCTTCAGCGCTTTGACTAGAATTTAAATAAAACGAAAG	
P142	CAGCAGTGCGTTGCTCTTGCT	To identify pJYS1-MAD7-TTTC-Δ*crtR*
P143	CAGTATCTCGAGCGAAGGCGA	
P144	AGTGATTGCTTATTCAGCACC	To clone pJYS1-MAD7-TTTC-Δ*crtEb*Δ*crtR*
P145	GCTGAATAAGCAATCACTGTCAAAAGACCTTTTTAATTTCTACTCTTGTAGATCC	

P146	ACACATGGGTATTCCTACACCCATAAAAC GAAAGGCCCAGTCTTTC	
P147	CGAAATTGGGCGTCTTGCTTATCGGCTGC GTCACCAAAAGTA	
P148	TTAACCTTGCACCCCATATGC	To identify pJYS1-MAD7-TTTC-Δ*crtEb*Δ*crtR*
P149	GCGCCATATACAACGATTGGGAA	
P150	GAAAGGAGGCCCTTCAGATGGAC	To clone pZ9-*crtE-crtB-crtI*
P151	CTGAAGGGCCTCCTTTCCTAAGATTTGCG GCTGGCTAGT	
P152	CCAGCCGCAAATCTTAGGAAAGGAGGCC CTTCAGATGACACACCAAAATTCGCCTCT	
P153	AGTTCCCTACTCTCGCATCCTTAATGATCG TATGAGGTC	
P154	GGATGCGAGAGTAGGGAACT	
P155	CTGAAGGGCCTCCTTTCTTGTTATCCGC	
P156	CTGCCGTTCGATGATCAAAACA	To identify pZ9-*crtE-crtB-crtI*
P157	TTACATGCCGATACCAGTGCAG	
P158	GGTCTCCGTCAATAACATCGT	
P159	CCAGCCGCAAATCTTAGGAAAGGAGGCC CTTCAGATGTCTACTAGCATCACAAC	To clone pZ9-*cg0722-crtB-crtI* and pZ9-*crtE-cg0722-crtB-crtI*
P160	GAATTTTGGTGTGTCATCTGAAGGGCCTCC TTTCTCATAGCTGAGGCTGCTTCT	
P161	GAAAGGAGGCCCTTCAGATGACACACCA AAATTCGCCTCT	
P162	TTGCCTTCATTGTCGCTTTTG	To identify pZ9-*cg0722-crtB-crtI*
P163	ATTTTGGCAGTTGAGCAACCA	
P164	TGCCTGCTTTGTTCTACGACAT	To identify pZ9-*crtE-cg0722-crtB-crtI*
P165	GATATTCAAGACAACTTCGGC	
P166	TTGTTATCCGCTCACAATTCC	To clone pZ9-*idsA-idi-crtB-crtI*
P167	TTGTGAGCGGATAACAAGAAAGGAGGCC CTTCAGATGAGCAGTTTCGATGCC	
P168	CTACATCCGACGTTCGGTTG	

P169	CAACCGAACGTCGGATGTAGGAAAGGAGGCCCTTCAGATGACTACTGAGGTTGAACTGG	
P170	CTGAAGGGCCTCCTTTCTTACTCTGCGTCAAACGCTTCC	
P171	GAAATTTGTCGAGGTCAAGGT	To identify pZ9-*idsA*-*idi*-*crtB*-*crtI*
P172	GTGGAAAACGCGAAATGCAGAGG	
P173	CTTTTAAGGAAGAGAGGCGAAT	

Table S3 Editing efficiency of large DNA fragment deletion in *C. glutamicum*.

	**crRNA**	**PAM**	**Efficiency**
Δ5 kb	TACTACACACCCATGACCTCT	TTTC	15/15;14/15;12/15
Δ20 kb	TACTACACACCCATGACCTCT	TTTC	7/8;9/13;11/14
Δ25 kb	TACTACACACCCATGACCTCT	TTTC	2/4;3/9;2/3
